# Evaluation of a three-gene methylation model for correlating lymph node metastasis in postoperative early gastric cancer adjacent samples

**DOI:** 10.3389/fonc.2024.1432869

**Published:** 2024-10-17

**Authors:** Shang Chen, Shoubin Long, Yaru Liu, Shenglong Wang, Qian Hu, Li Fu, Dixian Luo

**Affiliations:** ^1^ Guangdong Key Laboratory for Biomedical Measurements and Ultrasound Imaging, National-Regional Key Technology Engineering Laboratory for Medical Ultrasound, School of Biomedical Engineering, Shenzhen University Medical School, Shenzhen, China; ^2^ Laboratory Medicine Centre, Shenzhen Nanshan People’s Hospital, Shenzhen University, Shenzhen, China; ^3^ Hunan Provincial University Key Laboratory of the Fundamental and Clinical Research on Functional Nucleic Acid, Hunan Provincial Key Laboratory of the Traditional Chinese Medicine Agricultural Biogenomics, Changsha Medical University, Changsha, China; ^4^ School of the First Clinical Medical, Ningxia Medical University, Yinchuan, China; ^5^ Institute of Pharmacy and Pharmacology, School of Pharmaceutical Science, Hengyang Medical School, University of South China, Hengyang, China; ^6^ Guangdong Provincial Key Laboratory of Regional Immunity and Diseases, Department of Pharmacology and International Cancer Center, Shenzhen University Health Science Center, Shenzhen, China; ^7^ Department of Biomedical Engineering, Shenzhen University Medical School, Shenzhen, China

**Keywords:** early gastric cancer, paracancerous tissue, lymph node metastasis, DNA methylation, predictive model

## Abstract

**Background:**

Lymph node metastasis (LNM) has a profound impact on the treatment and prognosis of early gastric cancer (EGC), yet the existing evaluation methods lack accuracy. Recent research has underscored the role of precancerous lesions in tumor progression and metastasis. The objective of this study was to utilize the previously developed EGC LNM prediction model to further validate and extend the analysis in paired adjacent tissue samples.

**Methods:**

We evaluated the model in a monocentric study using Methylight, a methylation-specific PCR technique, on postoperative fresh-frozen EGC samples (n = 129) and paired adjacent tissue samples (n = 129).

**Results:**

The three-gene methylation model demonstrated remarkable efficacy in both EGC and adjacent tissues. The model demonstrated excellent performance, with areas under the curve (AUC) of 0.85 and 0.82, specificities of 85.1% and 80.5%, sensitivities of 83.3% and 73.8%, and accuracies of 84.5% and 78.3%, respectively. It is noteworthy that the model demonstrated superior performance compared to computed tomography (CT) imaging in the adjacent tissue group, with an area under the curve (AUC) of 0.86 compared to 0.64 (p < 0.001). Furthermore, the model demonstrated superior diagnostic capability in these adjacent tissues (AUC = 0.82) compared to traditional clinicopathological features, including ulceration (AUC = 0.65), invasional depth (AUC = 0.66), and lymphovascular invasion (AUC = 0.69). Additionally, it surpassed traditional models based on these features (AUC = 0.77).

**Conclusion:**

The three-gene methylation prediction model for EGC LNM is highly effective in both cancerous and adjacent tissue samples in a postoperative setting, providing reliable diagnostic information. This extends its clinical utility, particularly when tumor samples are scarce, making it a valuable tool for evaluating LNM status and assisting in treatment planning.

## Introduction

Early gastric cancer (EGC) is defined as lesions that are confined to the mucosa or submucosa, regardless of whether Lymph node metastasis (LNM) is present (1). Currently, the determination of LNM in preoperative evaluations of GC patients primarily relies on auxiliary examination methods (2), including gastroscopy, CT abdominal scans, endoscopic ultrasound, and tissue biopsy. Studies have revealed that only around 20% of EGC patients diagnosed with post-gastrectomy LNM based on pathological assessment are confirmed positive for LNM (3). Moreover, imaging techniques frequently encounter difficulties in differentiating nodules smaller than 2 cm as LNM, which may result in the administration of unnecessary treatments (4, 5). In cases where serum markers and imaging techniques fail to provide conclusive results, tissue biopsy for pathological identification becomes necessary. However, sampling nodules with unclear boundaries, proximity to large blood vessels, or multiple uncertain nodules poses significant challenges. Recent studies have highlighted the role of the precancerous lesions in tumor growth and metastasis initiation, offering insights into precancerous lesions for early diagnosis (6, 7). Peritumoral tissues, more readily accessible than tumor tissues, have emerged as valuable sources for predicting cancer staging and types based on molecular data. This information enhances understanding of tumor staging progression and facilitates the development of novel cancer diagnostic methods. To overcome the challenges in clinically differentiating EGC LNM, especially in cases where cancer tissues are not obtained or are limited in quantity, leveraging peritumoral tissue detection to assess the risk of LNM holds significant clinical promise.

DNA methylation represents a pivotal epigenetic modification, frequently observed in the early stages of cancer, and plays a significant role in cancer development and evolution. Its aberrations can serve as key indicators for early tumor detection (8–10). In previous studies, we developed an EGC LNM prediction model using the Epigenome-wide Profiling of DNA Methylation (EPIC) technique, which incorporates three genes (FCGBP, GNAS, CCDC166). This model demonstrated superior performance compared to conventional clinical methods, such as CT imaging and serum biomarker assessment, in predicting LNM (11). Recent investigations have identified a specific region at the interface between cancerous and adjacent tissues in liver cancer samples. When tumor cells invade, adjacent tissues often experience ischemic-hypoxic conditions, leading to a pronounced inflammatory and immunosuppressive microenvironment. This suggests that molecular processes and activities in adjacent tissues are closely linked to cancer characteristics and status, providing valuable insights into cancer type prediction, occurrence, progression, and staging (6, 7, 12). Consequently, in this study, we aim to further explore the diagnostic potential of the three-gene methylation model in both cancerous and adjacent tissues, assess its clinical utility, and propose it as a potential adjunctive diagnostic tool for clinical EGC LNM diagnosis.

## Materials and methods

### Clinical samples

**Figure 1:**
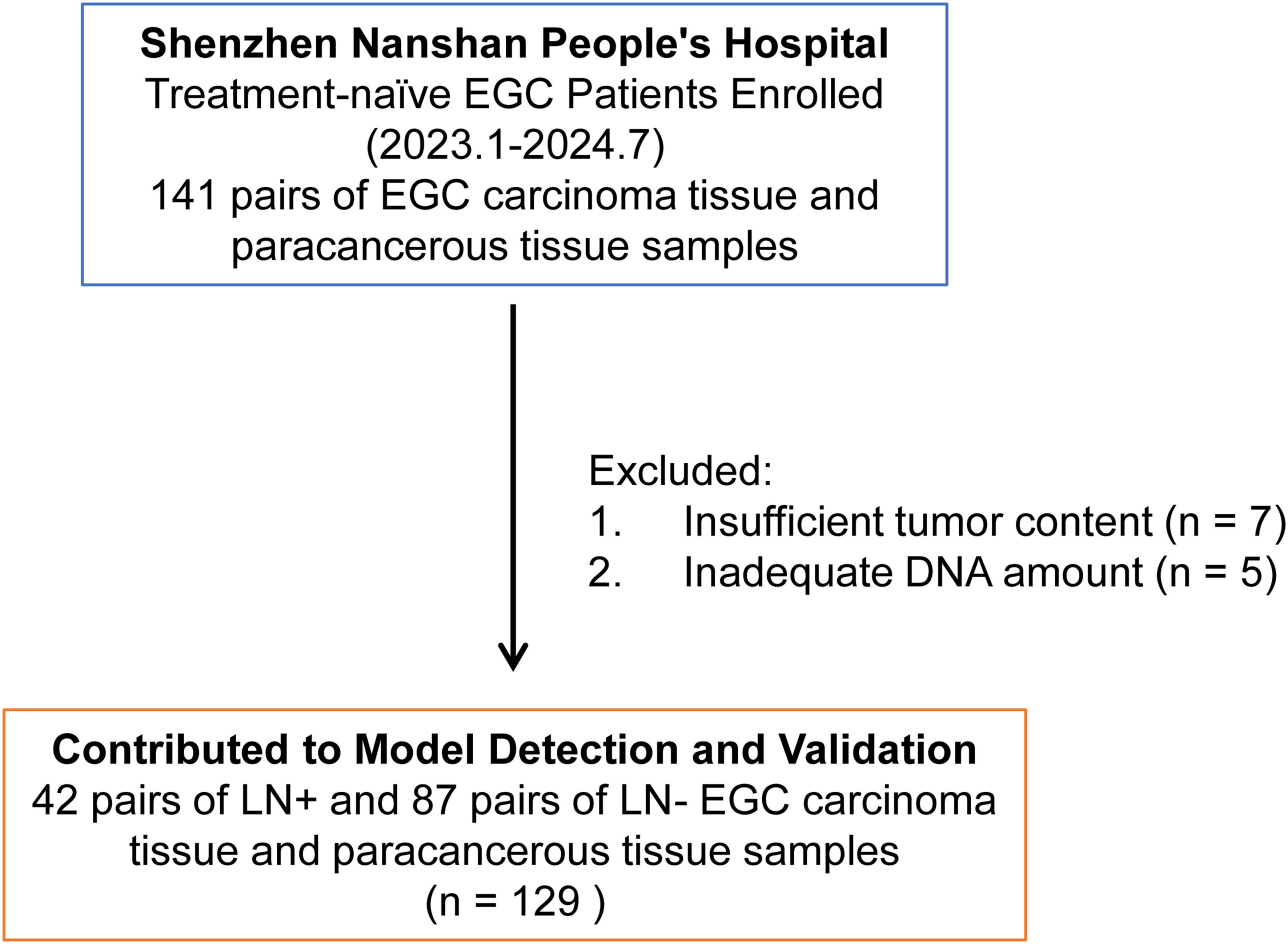
Overview of patient recruitment workflow. A total of 141 pairs of carcinoma tissue and paracancerous tissue samples were obtained from treatment-naïve early gastric cancer (EGC) patients at Shenzhen Nanshan People’s Hospital between January 2023 and July 2024. However, 12 samples were excluded from further analysis due to failed quality control (QC) in experimental procedures, specifically insufficient tumor content (n = 7) and inadequate DNA extraction from tumors (n = 5). The remaining 129 pairs of patient samples were subsequently used for model detection and validation.

Fresh frozen (FF) tissue samples of EGC were collected from January 2023 to July 2024 at Shenzhen Nanshan People’s Hospital. Inclusion criteria for EGC samples included: 1. Pathological diagnosis of T1 stage GC, 2. Absence of distant metastasis or family history of hereditary cancer, 3. Initial diagnosis of EGC without neoadjuvant therapy, and 4. Exclusion of lymphoma, multiple tumors, residual cancer, and intraepithelial neoplasia. The patient recruitment process is outlined in **Figure 1**. Untreated EGC patients (n=141, FF samples) were recruited from Shenzhen Nanshan People’s Hospital, during the same period. For surgical specimens, cancer tissues and paracancer tissues were collected, which aligns with that of previous research (13, 14). We include a detailed description of the selection criteria for adjacent tissues (**Supplementary Figure 1**): 1. Proximity to the tumor (within 5-20mm from the visible tumor margin). 2. Be free from tumor infiltration (may exhibit pathological alterations such as chronic inflammation, atrophy, intestinal metaplasia, or dysplasia). 3. Signs of inflammation or fibrosis. Twelve samples were excluded due to failed experimental quality control. A total of 129 pairs of cancer and adjacent tissue samples were included, comprising 42 pairs with lymph node metastasis-positive (LN+) tumor and 87 pairs lymph node metastasis-negative (LN-) samples. Tissue samples were obtained from surgical specimens before radiotherapy or chemotherapy. Clinical data, including age, sex, tumor characteristics, and pathological features, were collected. The association between clinical characteristics and LNM in 129 EGC cases was statistically analyzed using chi-square or Fisher’s exact test, as presented in **Supplementary Table 1**. All samples were from surgically resected tissues and pathologically diagnosed by at least two hospital pathologists. Tumor content exceeding 30% was confirmed in FF samples. This study was approved by the Ethics Committee of Shenzhen Nanshan People’s Hospital (Ethics Approval No.: KY-2023-035). Written informed consent was received from all participants.

### DNA extraction and bisulfite treatment

Samples were processed using the AllPrep DNA/RNA Mini Kit (Qiagen, Germany) according to the manufacturer’s instructions to extract genomic DNA from FF specimens. Genomic DNA was quantified with the Qubit dsDNA HS Assay Kit (Thermo Fisher Scientific, USA). Quality control criteria for EGC samples included DNA quantity greater than 100 ng and the main band of agarose gel electrophoresis larger than 500 bp. Bisulfite treatment was performed on each tissue sample using 50 ng of genomic DNA with the EZ-96-DNA Methylation Direct MagPrep Kit (Zymo Research, USA).

### Methylation analysis by qPCR

The methylation analysis utilized the MethyLight method as previously described (15). MethyLight assays were performed on the Quant Studio 3 Real-Time PCR System (Thermo Fisher, USA). The reaction followed thermal cycles consisting of initial denaturation at 98°C for 30 seconds, followed by 20 cycles of denaturation at 98°C for 15 seconds, annealing at 60°C for 15 seconds, and extension at 72°C for 15 seconds, with a final extension step at 72°C for 5 minutes. The resulting multiplex PCR products were used for quantifying targeted regions of interest. The qPCR reaction involved an initial denaturation at 95°C for 5 minutes, followed by 40 cycles of denaturation at 95°C for 15 seconds, annealing at 62°C for 1 minute, and fluorescence signal acquisition at 62°C. Co-methylation levels of a genomic region of interest were represented by ΔCt (cycle threshold), where ΔCt = Mean Ct (region of interest) - Mean Ct (region of control). The assay amplified methylated bisulfite-converted DNA fragments of regions of interest, with the resulting ΔCT values inversely correlated with the percentages of methylated molecules among total bisulfite-converted DNA molecules. The primers and probes used are shown in **Supplementary Table S2.**

### Development and evaluation of the traditional prediction model

The univariate analysis in the tumor tissues dataset included six clinicopathologic characteristics to explore their association with LNM. Variables with a p-value < 0.05 from this analysis were subsequently included in the multivariate analysis for the conventional model. Forward stepwise regression analysis was utilized to evaluate odds ratio (OR) values, accompanied by a 95% confidence interval (CI), in order to identify independent predictors. Multicollinearity of the multivariate models was assessed using tolerance and variation inflation factors. The quantitative scoring formula was derived from the coefficients and intercepts obtained through the multivariate logistic regression model. The area under the receiver operating characteristic curve (ROC) and the corresponding AUC values were calculated. The cutoff value of the model score was determined based on the maximum Youden index.

### Statistical analysis

The following R packages were utilized: pROC (1.16.1) for ROC and AUC calculations, ggplot2 (3.2.1) and RColorBrewer (1.1.2) for figure visualization, and glmnet (2.0.16) for logistic regression-based model construction. Univariate and multivariate logistic regressions were employed to assess the statistical significance of clinicopathological variables. AUC values were compared using the DeLong test. Sensitivity, specificity, and accuracy of both the 3-marker methylation model and the conventional model in detecting LNM were determined by comparison to pathology. Statistical analyses and data visualization were performed using R (3.6.0) and GraphPad Prism 8. A p-value < 0.05 on both sides of all hypothesis tests was considered statistically significant.

## Results

### The performance of a three-gene methylation model in postoperative EGC cancerous and paracancerous tissues

**Figure 2:**
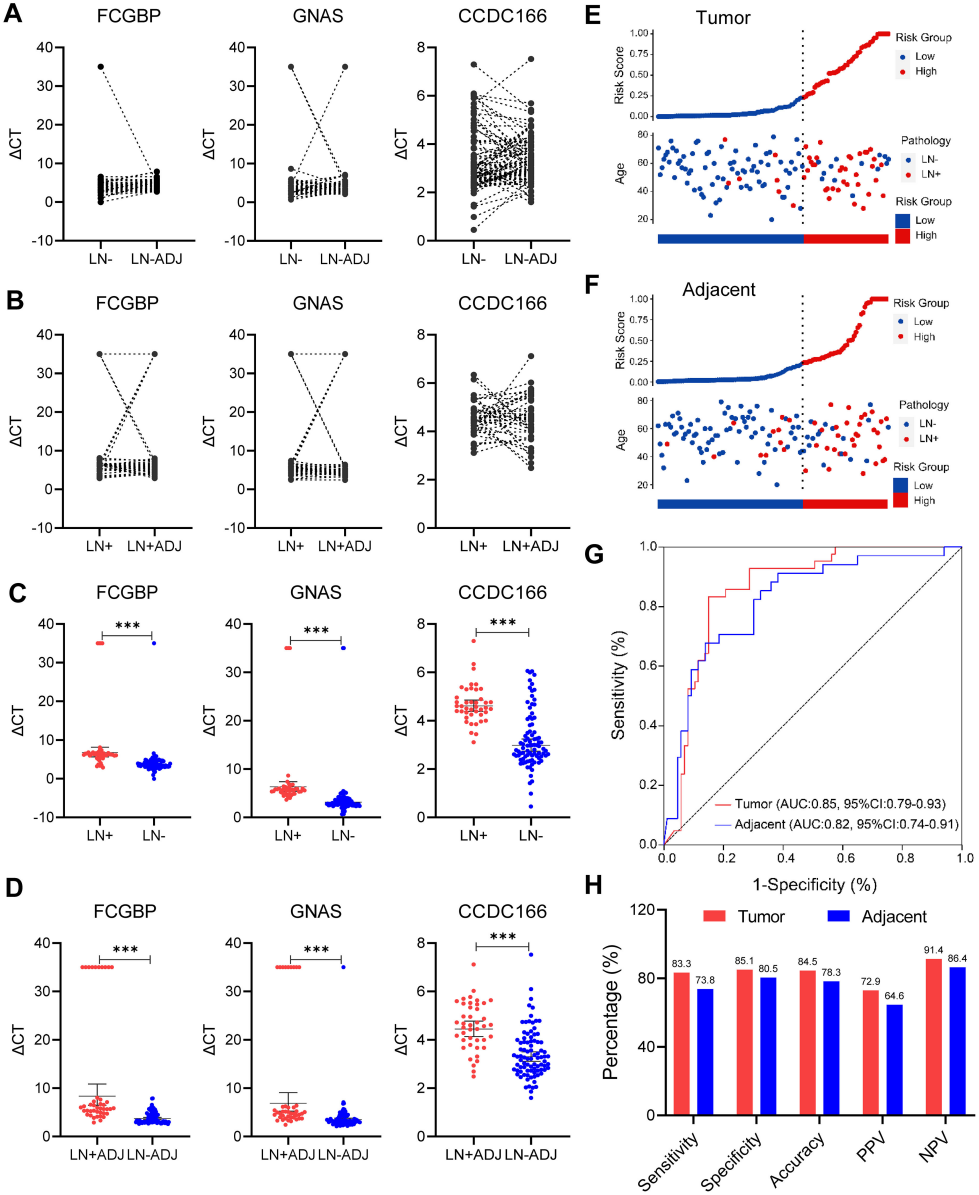
Evaluation of methylation model in the detection of LNM in EGC cancer and adjacent cancer. **(A)** Comparison of methylation levels of FCGBP, GNAS, and CCDC166 genes in EGC lymph node metastasis-negative (LN-) and paired adjacent samples (LN-ADJ) (paired T-test). **(B)** Comparison of methylation levels of FCGBP, GNAS, CCDC166 genes in matched adjacent samples of EGC lymph node metastasis-positive (LN+ vs. LN+ADJ, paired T-test). **(C)** Comparison of methylation levels of FCGBP, GNAS, and CCDC166 genes in EGC LN+ and LN- cancer tissue samples (T-test), error bars represent mean ± SD, ***p < 0.001. **(D)** Comparison of methylation levels of FCGBP, GNAS, and CCDC166 genes in EGC LN+ADJ and LN-ADJ samples (T-test), error bars represent mean ± SD, ***p < 0.001. **(E)** 3-gene methylation model for LN+ stratification in 129 EGC cancer samples. **(F)** 3-gene methylation model stratification of LN+ in 129 EGC adjacent samples. **(G)** ROC curves of a 3-gene methylation model in EGC cancer and adjacent samples. **(H)** Sensitivity, specificity, accuracy, positive predictive value (PPV), and negative predictive value (NPV) of the 3-gene methylation model (cutoff = 0.233) in carcinoma and adjacent samples, statistical significance was assessed by χ^2^ test.

A statistical analysis of clinical and pathological data from 129 cases of EGC revealed a significant association between lymphatic invasion and ulceration with LNM (P < 0.05), as illustrated in **Table 1**. In contrast, gender, age, tumor size and clinic serum tumor marker including carcinoembryonic antigen (CEA), Carbohydrate antigen 19-9 (GA19-9) and carbohydrate antigen 72-4 (CA72-4) did not exhibit statistically significant differences (P > 0.05). The MethyLight method was employed to assess the methylation levels of three genes (FCGBP, GNAS, and CCDC166) in paired peritumoral samples from 42 EGC LN+ cases and 87 LN- cases. A comparative analysis of the methylation levels, quantified by MethyLight data (ΔCT values), revealed no statistically significant differences for FCGBP, GNAS, and CCDC166 in both EGC LNM positive and negative samples, as well as their paired peritumoral samples (**Figures 2A, B**). However, the methylation levels of FCGBP, GNAS, and CCDC166 were found to be significantly higher in EGC LN+ tissue samples compared to EGC LN- tissue samples. It is noteworthy that the methylation levels of FCGBP, GNAS, and CCDC166 in LN+ paired peritumoral samples were significantly higher than those in LN- paired peritumoral samples (**Figures 2C, D**).

**Table 1:** Characteristics of EGC patients in 129 cases.

Characteristics	LN+, n = 42 (%)	LN-, n = 87, (%)	χ^2^	*p* value
Gender				
Man	23 (54.76)	57 (65.52)	1.139	0.238
Female	19 (45.24)	30 (34.48)		
Age				
≥ 60	14 (33.33)	38 (43.68)	1.260	0.262
< 60	28 (66.67)	49 (56.32)		
Tumor size				
>2cm	25 (59.52)	48 (55.12)	0.218	0.640
≤2cm	17 (40.48)	39 (44.88)		
LVI				
Presence	19 (45.24)	11 (12.65)	16.860	<0.0001***
Absence	23 (54.76)	76 (87.35)		
Invasional depth				
M	10 (23.81)	47 (54.08)	10.480	0.0012**
SM	32 (76.19)	40 (45.98)		
Ulceration				
Presence	25 (59.52)	34 (39.08)	4.770	0.029**
Absence	17 (40.48)	53 (60.92)		
Differentiation				
Differentiated	7 (16.67)	40 (45.98)	10.510	0.0012**
Undifferentiated	35 (83.33)	47 (54.08)		
CA19-9 (U/ml)				
>27	8 (19.05)	16 (18.39)	0.081	0.828
≤27	34 (80.95)	71 (81.61)		
CA72-4 (U/ml)				
>6.9	9 (21.43)	14 (16.10)	0.703	0.402
≤6.9	33 (78.57)	73 (83.90)		
CEA (ug/L)				
>5	12 (28.58)	19 (21.84)	0.486	0.485
≤5	30 (71.42)	68 (78.16)		
CT imaging				
Presence	14 (33.33)	15 (17.25)	4.209	0.040*
Absence	28 (66.67)	72 (82.75)		

LVI, lymphovascular invasion; CI, confidence interval; M, mucosa; SM, submucosa. *p<0.05, **p<0.01, ***p<0.001.

A quantitative scoring formula has been developed for predicting EGC LNM, which relies on a three-gene methylation prediction model. This model is represented by the following equation: logit (odds) = -9.46172 + 1.31131 × GNAS - 0.04684 × FCGBP + 0.65185 × CCDC166 (11). The formula was used to evaluate the risk of LNM stratification in 129 paired EGC tumor and adjacent non-tumor samples. A risk score cutoff value of 0.233 was employed to successfully differentiate EGC LN+ tumor and adjacent non-tumor samples (LN+ADJ, n = 42) from LN- tumor and adjacent non-tumor samples (LN-ADJ, n = 87) into distinct risk levels (**Figures 2E, F**). Among the 129 tumor samples, 7 cases with LN+ were classified as low-risk, and 13 case with LN- was categorized as high-risk (**Figure 2E**). Among the 129 adjacent non-tumor samples, 11 LN+ cases were predicted as low-risk, and 17 LN- case were predicted high-risk LNM (**Figure 2F**). Furthermore, the three-gene methylation model demonstrated superior diagnostic performance in both tumor and adjacent non-tumor samples, with area under the curve (AUC) values of 0.85 (95% CI: 0.79-0.93) and 0.82 (95% CI: 0.74-0.91), respectively (**Figure 2G**). The model demonstrated consistent superior performance in terms of sensitivity (83.3% vs. 73.8%), specificity (85.1% vs. 80.5%), and accuracy (84.5% vs. 78.3%) in the tumor and adjacent non-tumor datasets, respectively (**Figure 2H**). These findings suggest that the previously established three-gene methylation prediction model performs well diagnostically in both tumor and adjacent non-tumor samples.

### Three-gene methylation model outperforms CT imaging and tumor serum markers in postoperative EGC paracancerous tissues

**Figure 3:**
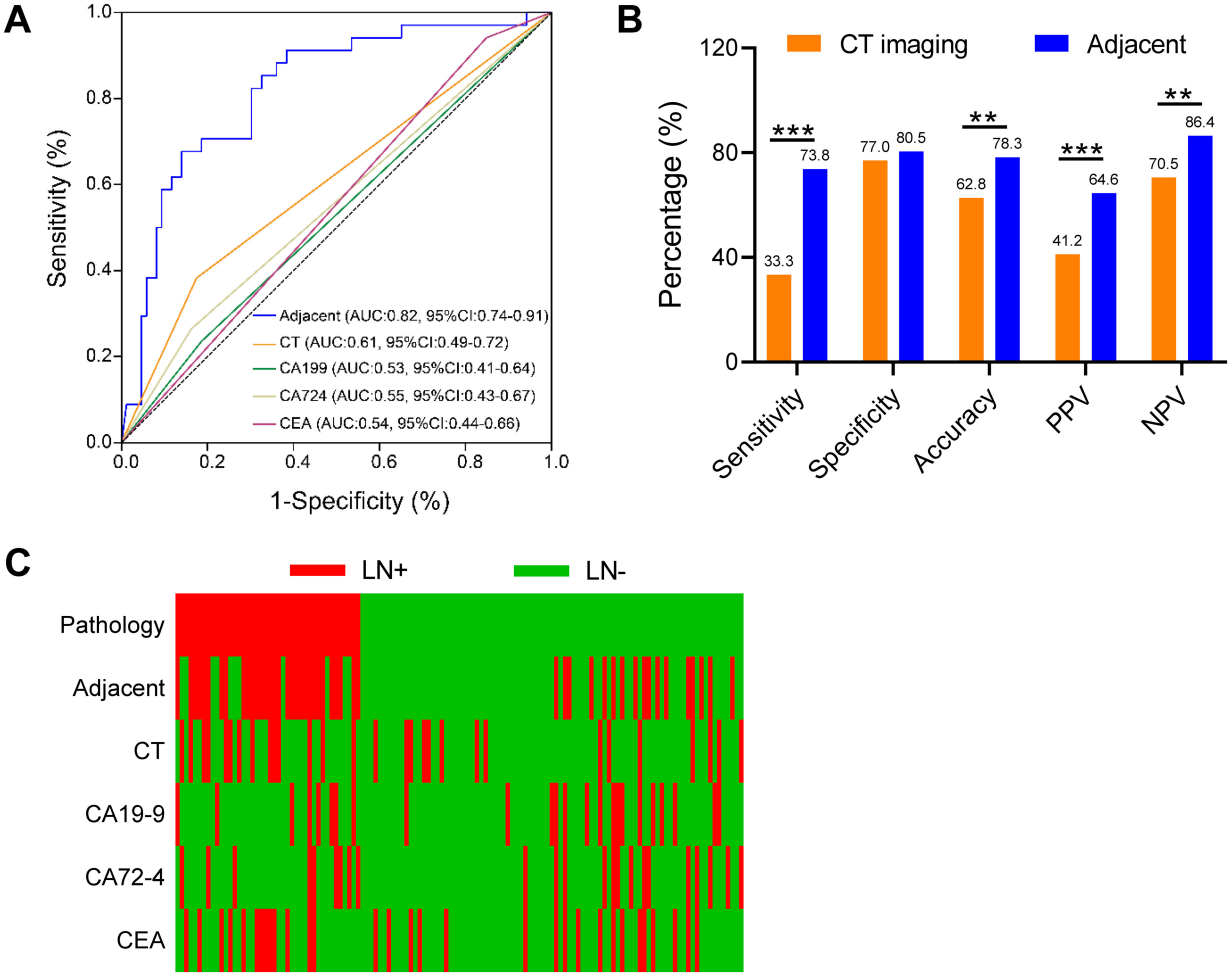
Performance of the three-gene methylation model compared to preoperative CT imaging and clinical serological tumor markers. **(A)** In adjacent cancer, the ROC curves of the 3-gene methylation model were compared to CT images and the ROC of clinical serological tumor markers including CA199, CA724, and CEA, respectively, and the comparison of AUC values was performed by the DeLong test. **(B)** Sensitivity, specificity, accuracy, positive predictive value (PPV), and negative predictive value (NPV) of the 3-gene methylation model and the conventional model in the adjacent cohort. **(C)** Distribution of LNM status in EGC predicted in carcinoma and adjacent cohorts, using 3-gene methylation model, CT images, CA199, CA724, and CEA, respectively. The χ2 test was statistically significant, **p<0.01, ***p<0.001.

In clinical practice, CT imaging is a commonly utilized tool for the detection of LNM and the determination of clinical stage N in patients with EGC. The diagnostic accuracy of CEA, CA19-9, CA72-4, and CT imaging for diagnosing LNM was evaluated. The diagnostic efficacy of the three-gene methylation panel for identifying LNM in EGC patients, as measured in adjacent tissues, was robust, with an area under the curve (AUC) of 0.82 (95% CI: 0.74-0.91). This performance significantly eclipsed that of conventional CT imaging, which had an AUC of 0.61 (95% CI: 0.49-0.72). Additionally, the AUCs for the biomarkers CA19-9, CEA, and CA72-4 were 0.53 (95% CI: 0.41-0.64), 0.54 (95% CI: 0.44-0.66), and 0.55 (95% CI: 0.43-0.67), respectively (**Figure 3A**, p < 0.001, Delong test). **Figure 3B** clearly demonstrates the superiority of the three-gene methylation model over CT imaging across multiple metrics, including specificity, sensitivity, accuracy, positive predictive value, and negative predictive value. Particularly noteworthy is the substantial increase in sensitivity, which nearly doubled, and the 15.7% improvement in accuracy. Although there was no change in specificity, the positive and negative predictive values saw respective increases of 23.4% and 15.9%. The three-marker methylation model demonstrated a high degree of concordance with clinicopathologic diagnoses of adjacent samples for patients with LN+ and LN- (**Figure 3C**). Therefore, the three-marker methylation model has the potential to assist in the avoidance of overtreatment in LN-patients.

### The three-gene methylation model is superior to traditional model based on clinicopathological characteristics

**Figure 4:**
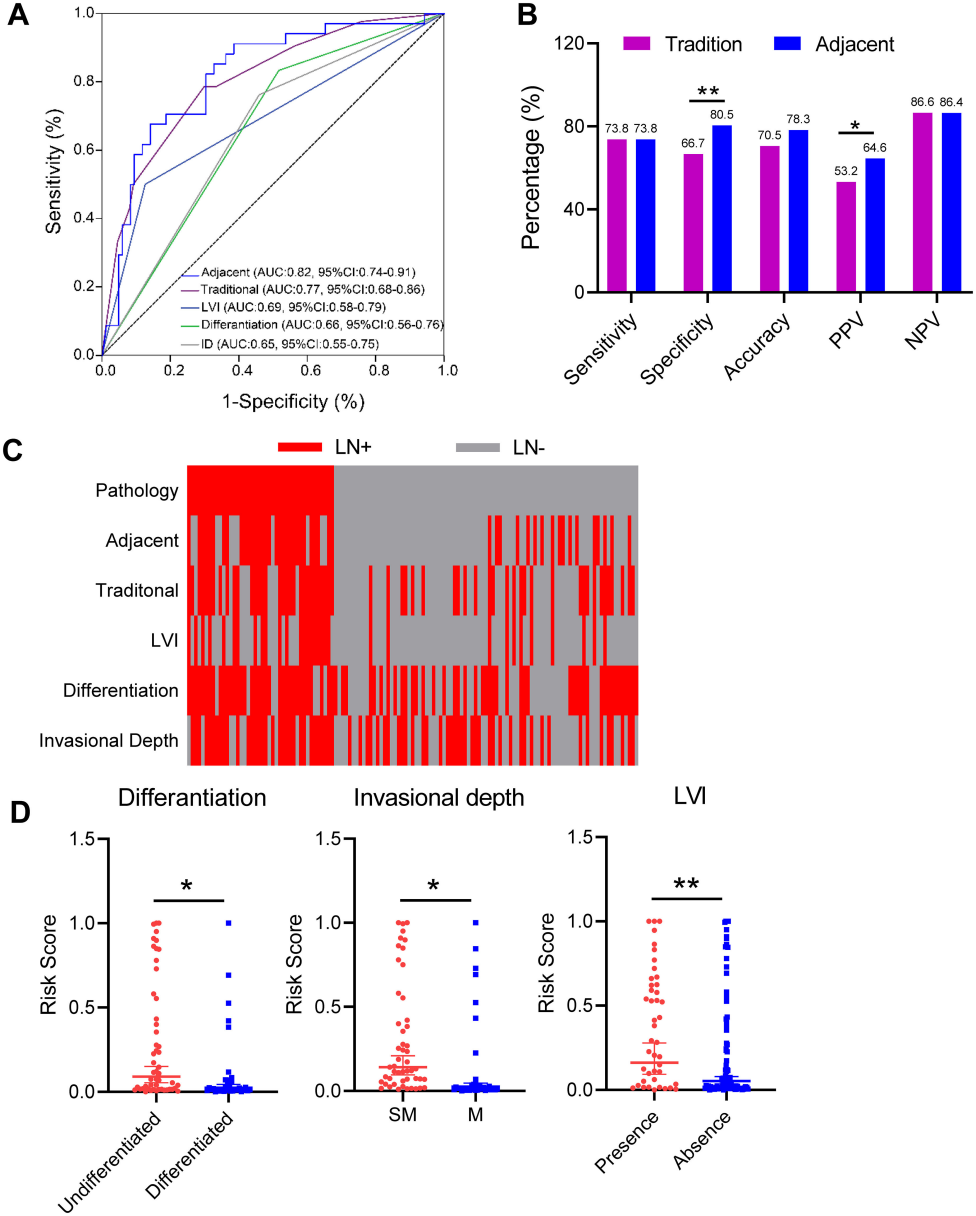
Performance of three-gene methylation models compared to traditional models and clinicopathological features. **(A)** The ROC curves of the 3-gene methylation model were compared to those of the conventional model and the ROC of clinicopathological features, including LVI, invasional depth and differentiation types, respectively, and the AUC values were compared by the DeLong test. **(B)** Sensitivity, specificity, accuracy, positive predictive value (PPV), and negative predictive value (NPV) of the 3-gene methylation model and the traditional model in the paracancerous cohort. Statistical significance was assessed by χ^2^ test. **(C)** Distribution of LNM status in EGC predicted in carcinoma and paracancerous cohorts, using 3-gene methylation models, traditional models, LVI, invasional depth (ID), and differentiation types, respectively. **(D)** LNM risk scores for 3-gene methylation models in the paracancerous cohort with different clinicopathologic features, including depth of infiltration, degree of differentiation, and LVI status. Error bars represent mean ± SD, *p < 0.05, **p < 0.01.

To evaluate the efficacy of the three-gene methylation model in comparison to the conventional preoperative clinicopathologic character-based model for the diagnosis of adjacent cancer, a univariate analysis was conducted on a sample of 129 cases. The analysis revealed significant associations between tissue differentiation (OR = 4.09, 95% CI: 1.49-11.20, p = 0.006), lymphovascular invasion (LVI) (OR = 4.89, 95% CI: 1.82-13.12, p = 0.002), invasional depth (OR = 3.18, 95% CI: 1.25-8.07, p=0.015), and CT imaging (OR = 2.41, 95% CI: 1.03-5.61, p = 0.043), and EGC LNM (**Table 2**). Subsequently, the meaningful indicators derived from the univariate analysis were incorporated into a multivariate logistic regression model using the stepwise backward method. A logistic regression (LR) method was employed to establish the traditional model. The results indicated that tissue differentiation (OR = 3.85, 95%CI: 1.43-10.37, p=0.008), LVI (OR = 5.71, 95% CI: 2.22-14.68, p<0.001), and invasional depth (ID) (OR = 3.37, 95%CI: 1.35-8.40, p=0.009) were independent risk factors for EGC LNM. Additionally, no evidence of multicollinearity was observed among the clinical variables included in the traditional model (**Supplementary Table S3**). A quantitative scoring formula for the traditional model was established based on the multivariate analysis. This formula is as follows: -2.909 + 1.348 × Differentiation + 1.742 × LVI + 1.215 × ID. In this dataset, the traditional model achieved an AUC of 0.77 (95% CI: 0.68–0.86), which is consistent with the results reported in previous studies (6, 7). Furthermore, in the cohort of 129 cases, the diagnostic efficacy of the three-gene methylation model for adjacent cancer (AUC = 0.82, 95% CI: 0.74–0.91) was markedly superior to that of individual clinicopathologic factors, such as invasional depth (AUC = 0.65, 95%CI: 0.55–0.75; p<0.001), differentiation (AUC = 0.66, 95% CI: 0.56–0.76; p<0.001), LVI (AUC = 0.69, 95% CI: 0.58–0.79; p < 0.001), and the traditional model (AUC = 0.77, 95% CI: 0.68–0.86, p=0.192) (**Figure 4A**). In comparison to the traditional model, the 3-marker methylation model exhibited markedly elevated specificity (80.5% vs. 66.7%), accuracy (78.3% vs. 70.5%), and positive predictive value (64.6% vs. 53.2%) in the adjacent cohort (**Figure 4B**). **Figure 4C** illustrates the predictive results of the three-gene methylation model, the traditional model, and associated clinical features. It is noteworthy that the 3-marker methylation model demonstrated superior consistency with clinicopathological diagnosis on adjacent samples in patients with LN+ and LN- when compared to the traditional model and related clinical features. Furthermore, the correlation between the risk score and clinical features was evaluated, demonstrating a markedly elevated LNM risk score in patients with ulceration, undifferentiated tumors, and LVI in the adjacent cohort (**Figure 4D**). This suggests a potential association between the LNM risk score and established independent risk factors for LNM. In conclusion, the three-gene methylation model demonstrated accurate and robust performance in identifying LNM in the paratumoral area of EGC.

**Table 2:** Univariate and multivariate logistic regression of LNM in 129 cases.

Characteristics		Univariate analysis			Multivariate analysis		
		OR	95%CI	*p*	OR	95%CI	*p*
**Tumor Size**	≥20mm	1.09	0.45-2.76	0.845			
**Differenciation**	G3	4.09	1.49-11.20	0.006	3.85	1.43-10.37	0.008
**Invasional depth**	SM	3.18	1.25-8.07	0.015	3.37	1.35-8.40	0.009
**Ulceration**	Presence	1.64	0.66-4.07	0.291			
**LVI**	Presence	4.89	1.82-13.12	0.002	5.71	2.22-14.68	<0.001
**CT imaging**	Presence	2.41	1.03-5.61	0.043			

OR, odds ratio; CI, confidence interval; M, mucosa; SM, submucosa; LVI, lymphovascular invasion.

## Discussion

The presence of LNM in patients with EGC is significantly associated with a poor prognosis. This finding is supported by two meta-analyses (15, 16). However, among the 356 patients who underwent additional gastrectomy, only 5% were found to have LNM, resulting in over 90% of patients receiving an inappropriate level of treatment. This resulted in a range of post-gastrectomy complications, including gastric bleeding, gastrojejunostomy leak, delayed gastric emptying, reflux esophagitis, and decreased postoperative quality of life. It is therefore imperative to accurately predict LNM in EGC patients in order to select the optimal treatment, avoid overtreatment, improve postoperative survival quality, and reduce economic losses. Nevertheless, there is currently no accurate method for predicting LNM in EGC.

Although CT imaging is a commonly utilized method for clinically assessing LNM, its accuracy in diagnosing LNM in GC is only 62% (17). In recent years, molecular biomarkers, including vascular endothelial growth factor (VEGF), coronin-like actin-binding protein 1C (CORO1C), epithelial cadherin (E-cadherin), and Ring Finger Protein 180 (RFP 180), have emerged as a prominent area of research in the context of GC LNM. These biomarkers are involved in the various stages of GC LNM and serve as markers for advanced stages of LNM (18–20). Nevertheless, there is a paucity of research examining the potential of DNA methylation strategies for diagnosing LNM in EGC. Wu et al. constructed 14 LNM-related gene classifiers using GC methylation data from The Cancer Genome Atlas (TCGA) to assess LNM risk in GC (21). In a previous study, we constructed an EGC LNM prediction model comprising three genes (FCGBP, GNAS, and CCDC166) using EPIC. We assessed and confirmed the superior predictive performance and clinical potential of the three-gene model for LNM in EGC, utilizing 425 formalin-fixed paraffin-embedded (FFPE) samples (11). Building on this, our current research incorporates an analysis of 129 postoperative frozen EGC samples. Additionally, we procured 129 pairs of tumor and adjacent non-tumor samples from EGC patients for this study. The model demonstrated excellent performance in cancer samples (AUC=0.85) and non-cancerous samples (AUC=0.82), exhibiting comparable accuracy in both categories. Moreover, the model demonstrated superior performance in non-cancerous samples compared to CT imaging (AUC=0.61) and the traditional model based on clinical pathological data (AUC=0.77).

The majority of previous studies have concentrated on tumor tissues with the objective of identifying the characteristics associated with cancer initiation and progression. Nevertheless, an increasing body of research indicates a strong correlation between the tumor microenvironment (TME) and the initiation and progression of tumors. The TME represents the site at which tumor growth and metastasis initiation occur. As tumors advance to a certain stage, adjacent non-cancerous tissues undergo ischemia and hypoxia, which foster chemotherapy resistance, cancer recurrence, and metastasis, thus resulting in a poor prognosis (22, 23). The adjacent tissue samples analyzed in this study were carefully selected from circumferential areas within 5-20 mm of the visible tumor margin. This specific range was chosen based on evidence suggesting that molecular alterations related to TME are likely to extend into this peritumoral area (13, 14). The selected adjacent tissues were non-tumorous but often exhibited pathological alterations such as chronic inflammation, atrophy, intestinal metaplasia, or dysplasia (24, 25). These changes are indicative of the tissue’s involvement in the TME which plays a critical role in the progression of EGC. In line with previous studies, the adjacent tissues in this study were found to frequently exhibit vascular changes consistent with ischemic or hypoxic conditions, which are common in the TME surrounding gastric cancers (26, 27). These conditions are known to drive significant molecular and epigenetic changes, including the methylation of genes involved in tumor promotion and metastasis. The molecular events and dynamics within the TME are likely correlated with the characteristics and progression of cancer. This correlation could provide valuable insights for predicting cancer type and stage. Wang et al. have documented the activation of complement and angiogenesis pathways within the TME, which are associated with the advancement of cancer (28). Recently molecular investigations have indicated that the recurrence of liver cancer following resection can be predicted not only by the genetic features of tumor tissues but also by those of adjacent non-cancerous tissues, including genes associated with immune responses (29). Evan et al. demonstrated the significance of the Wnt/TGF-β proliferative signaling pathway and immune inhibitory molecular characteristics in adjacent non-tumor tissues. From a clinical perspective, acquiring adjacent non-cancerous tissues is a more feasible undertaking than obtaining tumor tissues (30). From a clinical perspective, it is more straightforward to obtain adjacent non-cancerous tissues than it is to acquire tumor tissues. Consequently, the prediction of cancer staging and typing based on molecular data from adjacent non-cancerous tissues can provide valuable insights into the understanding of tumor staging progression and contribute to the development of novel approaches for cancer diagnosis.

The three-gene methylation model features FCGBP (IgG Fc binding protein), which has been demonstrated to enhance cancer infiltration and metastasis. FCGBP is significantly correlated with patient survival and prognosis across various cancers, including colorectal cancer, lung cancer, esophageal cancer, and glioma (31, 32). GNAS (G protein alpha subunit) functions as a crucial transduction protein capable of activating the Wnt/β-catenin and Hedgehog signaling pathways, thereby influencing the onset and progression of cancer (33, 34). The Coiled-Coil Domain-Containing (CCDC) family includes CCDC116, which has been identified as a member implicated in regulating crucial signaling pathways and genes (e.g., PI3K/AKT, ERK/RAS, c-Myc) that are essential for tumor growth, invasion, and metastasis. Consequently, CCDC116 influences cancer prognosis (35, 36). These three gene molecules, which are integral to the model, exhibit characteristics associated with cancer, including tumorigenesis and tumor progression. They may also play a pivotal role in the process of EGC LNM in adjacent tissues, which highlights the utility of our model. Ma et al. employed adjacent tissue DNA methylation data to identify methylation molecular features in staged cancers, including renal cancer, colorectal cancer, and liver cancer, with accuracies exceeding 0.72 (7). Although our study encompasses a range of cancer types, the DNA methylation profile of adjacent tissues has the potential to serve as a diagnostic and personalized targeted therapy tool, offering an alternative approach when accessing tumor tissue is challenging. Our study, while significant, is based on a modest sample of postoperative resections in both cancerous and adjacent tissue samples. While these findings suggest potential clinical utility, the model’s predictive value in a pre-operative context has being established. The promising correlation observed in this retrospective analysis warrants further investigation using pre-operative biopsy samples. Future studies should focus on standardizing the criteria for selecting adjacent tissues in biopsies, potentially guided by imaging techniques or specific histological markers, and conducting multicenter trials to validate the model’s robustness across diverse patient populations and pre-operative conditions. Such an approach is essential to confirm the robustness and effectiveness of our analytical methods.

In conclusion, our study proposes the potential of the EGC LNM three-gene methylation prediction model that we developed for use in cancer diagnosis, both for tumor tissue and adjacent tissues, as a promising potential tool for predicting LNM in EGC, particularly in situations where obtaining tumor tissue is difficult. However, its utility as a pre-operative predictive tool remains to be validated. Future research should aim to assess this model’s applicability to pre-operative biopsy samples, which would significantly enhance its clinical relevance. Moreover, the three-gene methylation prediction model exhibits substantial diagnostic advantages for assessing the likelihood of LNM in adjacent specimens, thereby enhancing the clinical applicability of this model and establishing a theoretical foundation for its utilization in clinical diagnosis.

## Data Availability

The authors declare that all data supporting the results in this study are available in the paper and **Supplementary Material**. Source data are available from the corresponding authors upon reasonable request.
