# Supplementary material for: Evaluation of a three-gene methylation model for correlating lymph node metastasis in postoperative early gastric cancer adjacent samples
Source: Front Oncol. 2024 Oct 17;14:1432869. doi: 10.3389/fonc.2024.1432869 (PMC11524798; doi:10.3389/fonc.2024.1432869)
Supplement: Supplementary file 1 [file DataSheet1.docx]

**Evaluation of a Three-Gene Methylation Model for Correlating Lymph Node Metastasis in Postoperative Early Gastric Cancer Adjacent Samples**

Shang Chen^1,2,3^, Shoubin Long^2^, Yaru Liu^2,4^, Shenglong Wang^2,4^, Qian Hu^2,5^, Li Fu^6^, Dixian Luo^2,4,7*^

**Supplementary Figures**


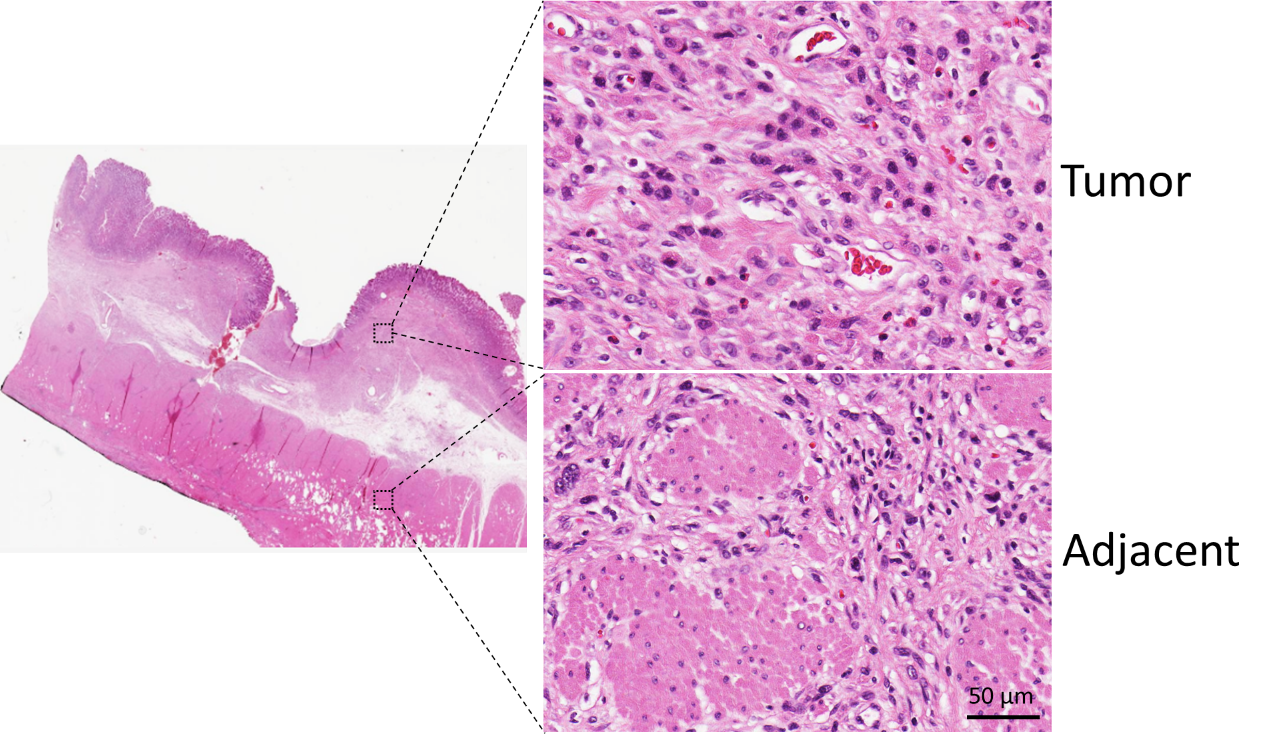


Supplementary Figure 1. Representative early gastric cancer pathological section with shematoxylin and eosin staining. Representative cancerous and adjacent sections of gastric cancer. Scale bar = 50 μm.
